# 2.L. Workshop: Public Health Institution and Academia’s role in building capacity for Health Impact Assessment

**DOI:** 10.1093/eurpub/ckac129.099

**Published:** 2022-10-25

**Authors:** Organised by: EUPHA-HIA, EUPHA-HWR, ASPHER, Chair persons: Piedad Martin-Olmedo (EUPHA-HIA), Ellen Kuhlmann (EUPHA-HWR)

## Abstract

Health impact assessment (HIA) is a process, which systematically judges the potential, and sometimes unintended effects of any new proposal (policy, project, program or strategy) on the population health, and the distribution of those effects within the population. It was conceived in 1999 as the supporting tool for the implementation of Health in All Policies (HiAP), but its practical use remains quite partial worldwide, with exceptional regions and countries (e.g. Wales (UK), Australia). Several surveys conducted nationally, but also across Europe by the World Health Organization-Regional Office for Europe identified several barriers that hinder a broader and more extensive practical application of HIA. One of these limitations lies in the lack of understanding and knowledge of professionals from non-health sectors about the benefits that addressing health considerations in the planning process of their projects, strategies or programs may have. In this sense, the HIA is frequently seen as an additional bureaucratic burden. Other common constraints reported refer to inadequate resources (guidelines, tools, evidence) and lack of qualified staff experienced in conducting HIA. Respondents from those surveys also deplored a history of unsatisfactory experiences involving intersectoral collaboration with health professionals, decision makers and other public sector stakeholders. Health agencies, such as national and regional Public Health Institutes (PHIs) can play a critical role in overcoming these limitations. They can act by providing independent advice and support based on the best available evidence to governments and proposal developers (projects, programs, strategies). However, they need to have knowledge, resources and capacity to do so. On the other hand, these basic concepts and procedures related to HiAP or HIA are rarely addressed in depth in the university curricular training of health sectors or other sectors such as environmental science or urbanism. Present workshop intends to analyse different experiences in Europe regarding the role of PHIs and also the academia in overcoming those reported exiting barriers, and reach a more sustainable and healthy societies. The fundamentals of what could be a training program on HIA principles across Europe will be also discussed.

**Key messages:**

• PHIs can demonstrate leadership for HIA and HiAP by providing support to decision makers in better understand the wider health impacts and how they will manifest themselves across the population.

• HIA practice requires of the design of proper training programs and of certain validation process, which avoids conflicts regarding consultants’ impartiality and independence in conducting a HIA.

